# The Power of Heterogeneity: Parameter Relationships from Distributions

**DOI:** 10.1371/journal.pone.0155718

**Published:** 2016-05-16

**Authors:** Magnus Röding, Siobhan J. Bradley, Nathan H. Williamson, Melissa R. Dewi, Thomas Nann, Magnus Nydén

**Affiliations:** 1 SP Food and Bioscience, Soft Materials Science, Göteborg, Sweden; 2 Future Industries Institute, University of South Australia, Adelaide, Australia; 3 ARC Centre of Excellence in Convergent Bio-Nano Science and Technology, Ian Wark Research Institute, University of South Australia, Adelaide, Australia; 4 MacDiarmid Institute, Victoria University of Wellington, Wellington, New Zealand; 5 School of Energy and Resources, UCL Australia, University College London, Adelaide, Australia; University of Cambridge, UNITED KINGDOM

## Abstract

Complex scientific data is becoming the norm, many disciplines are growing immensely data-rich, and higher-dimensional measurements are performed to resolve complex relationships between parameters. Inherently multi-dimensional measurements can directly provide information on both the distributions of individual parameters and the relationships between them, such as in nuclear magnetic resonance and optical spectroscopy. However, when data originates from different measurements and comes in different forms, resolving parameter relationships is a matter of data analysis rather than experiment. We present a method for resolving relationships between parameters that are distributed individually and also correlated. In two case studies, we model the relationships between diameter and luminescence properties of quantum dots and the relationship between molecular weight and diffusion coefficient for polymers. Although it is expected that resolving complicated correlated relationships require inherently multi-dimensional measurements, our method constitutes a useful contribution to the modelling of quantitative relationships between correlated parameters and measurements. We emphasise the general applicability of the method in fields where heterogeneity and complex distributions of parameters are obstacles to scientific insight.

## Introduction

Increasing the dimensions of an experiment allows the measurement of dependence between parameters. For example, introducing multi-dimensional nuclear magnetic resonance (NMR) has opened new possibilities for studying heterogeneous structures and complex phenomena by correlating different parameters describing transport properties and identifying different populations based on diffusion and relaxation properties [1, 2]. In optical (electronic) spectroscopy in the infrared, visible, and ultraviolet ranges, obtaining 2D spectra that describe correlations of excitation and detection wavelengths recently enabled the mapping of energy transfer pathways in the light-harvesting mechanism of certain bacterial species [3–5]. The key notion for both techniques is that a *joint* probability distribution (or ‘spectrum’ or ‘map’) of different parameters and their relationships can be estimated, instead of just the *marginal* (or lower-dimensional) distributions for individual parameters.

Multi-dimensional *intra*-modality measurements—within the confines of a single technique—inherently provide probabilistic relationships between parameters. More often than not, though, different parameters of systems are studied using different techniques, i.e. a multi-dimensional *inter*-modality measurement; revealing parameter relationships then becomes a matter of data analysis post-experiment, rather than a matter of experiment per se. Unfortunately, individual parameters in almost all real systems are distributed, and heterogeneity is a major obstacle to data interpretation. One common approach to this problem is to rely on purification to minimise sample heterogeneity, estimating relationships using standard methods such as regression by least-squares fitting. Altering samples is typically not viable in the industrial processing of heterogeneous raw materials, and this can be a substantial hurdle even in controlled laboratory settings.

This dilemma has prompted us to move to a setting where experimental input and output originate from different measurement techniques and comprise distributions rather than single values. Combining information from different sources, often called data integration or data fusion [6], is broadly addressed in computer science, machine learning, and mathematics. Modelling probabilistic relationships when data is obtained as distributions of individual parameters is almost unexplored; the closest work has discussed ‘distribution to distribution regression’ in a different context [7].

Here we develop a widely applicable general-purpose statistical method for estimating probabilistic relationships from distributions of data for individual parameters. Critically, these distributions can be represented by different types of data, i.e. statistical samples (sets of values), decay curves, spectra, function curves, and histograms, and therefore involve solving inverse problems.

## Results and Discussion

Consider the probabilistic relationship between an input parameter *x* and an output parameter *y* by modelling the joint distribution describing the dependence structure of *x* and *y*, based on measurements from *K* samples. The data from the *k*th sample represents marginal distributions *p*_*x*;*k*_(*x*) and *p*_*y*;*k*_(*y*) (directly as a statistical sample, or indirectly through an inverse problem) from the joint distribution for the *k*th sample, *p*_*x*, *y*;*k*_(*x*, *y*). Generally, nothing can be said about the dependence structure of *x* and *y* from a single measurement alone: finding a joint distribution from marginal distributions is by itself an inverse problem that requires assumptions or a priori knowledge. However, assuming that the conditional distribution of *y* given *x* is common to all samples, the joint distribution of *x* and *y* for the *k*th sample can be written *p*_*x*, *y*;*k*_(*x*, *y*) = *p*_*y*|*x*_(*x*, *y*)*p*_*x*;*k*_(*x*). Hence,

$$p_{y;k}\left(y\right)=\int_{0}^{\infty }p_{y|x}\left(x,y\right)p_{x;k}\left(x\right)\;\text{d}x.$$ (1)

A ‘link’ between the measurements for different samples is provided through the conditional distribution *p*_*y*|*x*_(*x*, *y*), interpreted physically as the distribution of *y* caused by unobserved parameters of the system other than *x*. That it is independent of sample index *k* corresponds to assuming that sorting into *K* samples is conducted solely on the basis of the value of *x*. The conditional distribution *p*_*y*|*x*_(*x*, *y*) is estimated indirectly by fitting the distributions *p*_*y*;*k*_(*y*) to the measurements, yielding an estimate of the probabilistic relationship between *x* and *y*. Thus, a space of distributions of *x* is mapped onto a space of distributions of *y*, in the process of modelling the relationship. The distribution models may be parametric as well as nonparametric.

First, we study probabilistic relationships between material parameters for colloidal semiconductor quantum dots (QDs), which are luminescent nanoparticles with applications in imaging [8–10], catalysis [11], photovoltaics [12–14], and many more fields. QDs comprise a prototypical complex material with properties dependent on the interaction between multiple parameters. In particular, the emission spectral profile and excited-state decay dynamics and their relation to size are vital for understanding the electro-optic and catalytic properties of these materials [15]. Additionally, QD size polydispersity is often inherent to synthesis and further purification to reduce the polydispersity is often impractical. A set of four batches of standard ZnS-coated CdSe QDs are synthesised and diameter distributions estimated from transmission electron microscopy (TEM) image data. Steady-state emission spectra of the QDs are acquired and interpreted as distributions of wavelengths *p*_λ;*k*_(λ) convolved with a Lorentzian line-broadening function, *γ*/(*π*((λ − λ′)^2^+*γ*^2^)). The model for the *k*th spectrum is therefore of the form

$$I_{k}\left(\lambda \right)=I_{0;k}\int_{0}^{\infty }p_{\lambda ;k}\left(\lambda^{\prime }\right)\frac{\gamma /\pi }{{\left(\lambda -\lambda^{\prime }\right)}^{2}+\gamma^{2}}\;\text{d}\lambda^{\prime }.$$ (2)

The distribution *p*_λ;*k*_(λ) is the marginal distribution of λ from the joint distribution

$$p_{d,\lambda ;k}\left(d,\lambda \right)=p_{\lambda |d}\left(d,\lambda \right)p_{d;k}\left(d\right).$$ (3)

**Fig 1:**
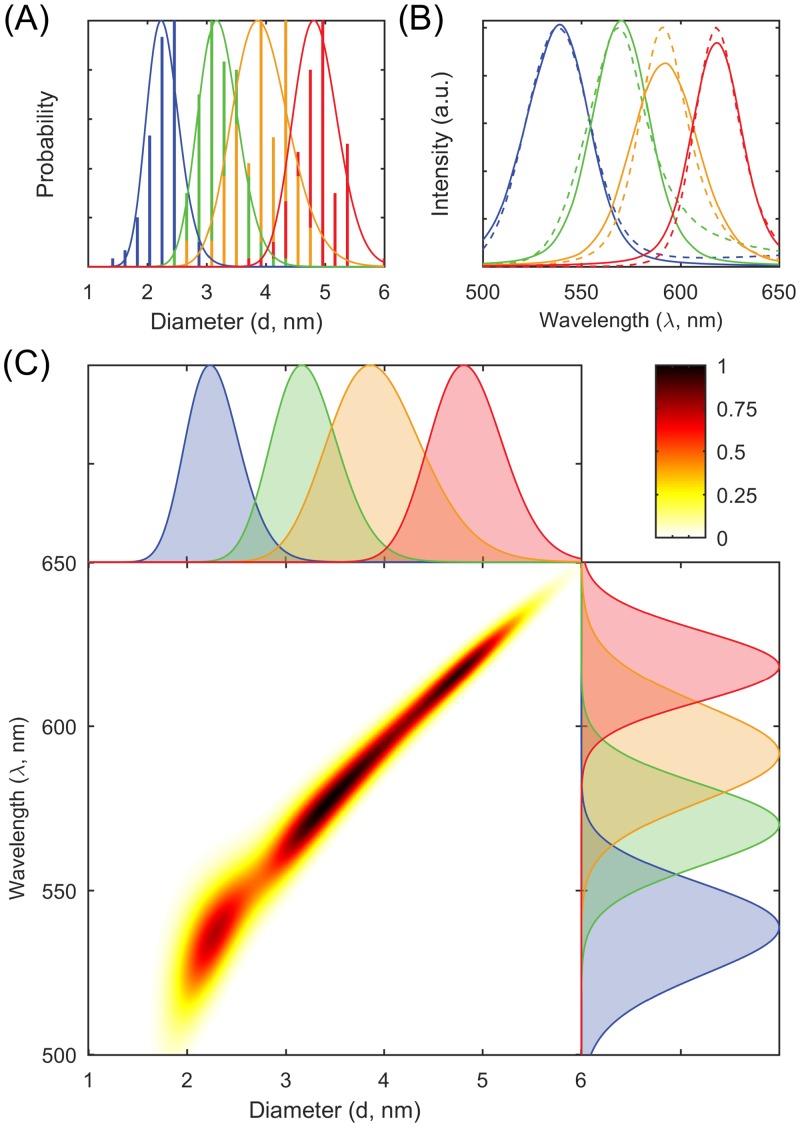
Correlating diameters to emission wavelengths. (A) Diameter histograms (*n* = 95, 127, 88, and 106) for the four samples (blue, green, yellow, and red) extracted from transmission electron microscopy and the model fits (solid lines). (B) Steady-state emission spectra (dashed lines) and the model fits (solid lines) for the four samples (blue, green, yellow, and red). (C) Estimated joint distributions of diameters and wavelengths with marginal diameter distributions at the top and marginal wavelength distributions at the right.

This factoring corresponds to assuming that the QDs are sorted only according to size, and that other variation for a given diameter *d* is the same regardless of the sample. The distribution *p*_λ|*d*_(*d*, λ) constitutes a summary of the influence of all system parameters other than *d*. If it is degenerate, *p*_λ|*d*_(*d*, λ) = *δ*(λ − λ_0_), λ is completely determined by *d*, and thus there is no other relevant parameter in the relationship. Fig 1 shows the spectra, the model fits, and the estimated joint distributions of diameters and emission wavelengths.

We now acquire fluorescence lifetime distributions of the QDs and interpret them as superpositions of exponential lifetime distributions, governed by a ‘characteristic’ (average) lifetime distribution for the *k*th sample, *p*_*τ*;*k*_(*τ*), such that

$$p_{t,k}\left(t\right)=\int_{0}^{\infty }p_{\tau ;k}\left(\tau \right)\frac{1}{\tau }\text{exp}\left(-\frac{t}{\tau }\right)\;\text{d}\tau .$$ (4)

The distribution *p*_*τ*;*k*_(*τ*) is the marginal distribution of *τ* coming from the joint distribution

$$p_{d,\tau ;k}\left(d,\tau \right)=p_{\tau |d}\left(d,\tau \right)p_{d;k}\left(d\right).$$ (5)

**Fig 2:**
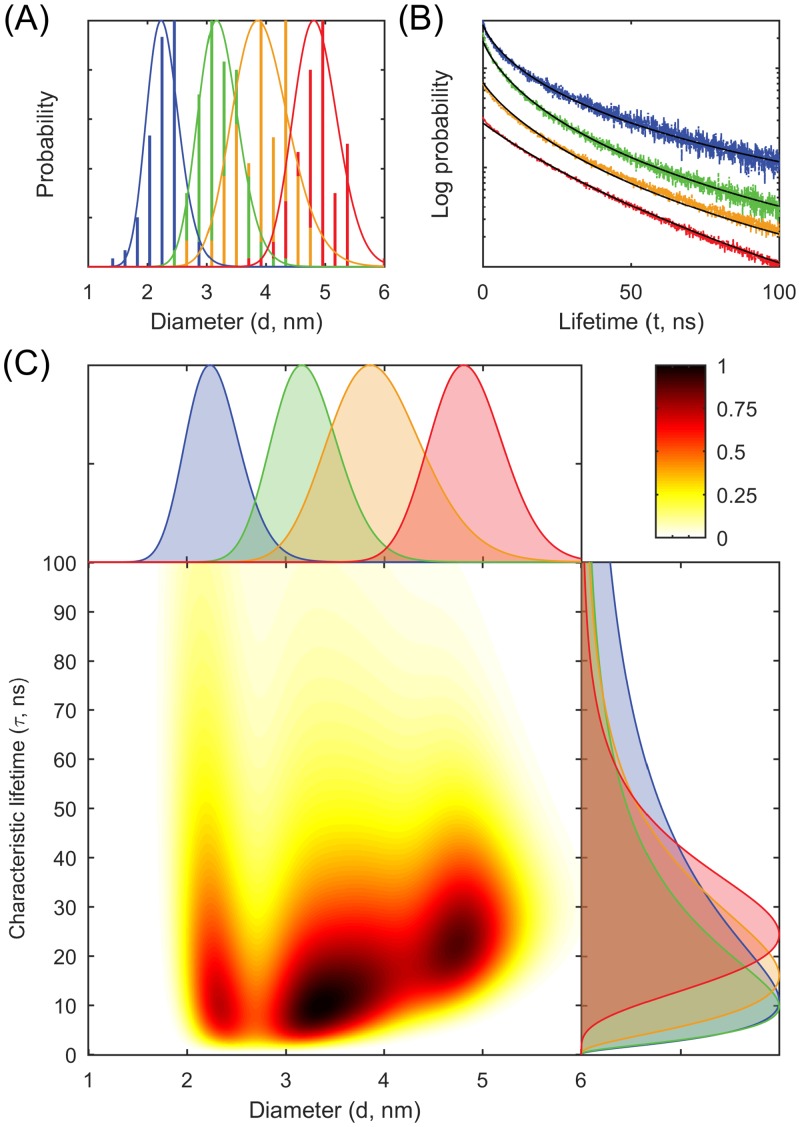
Correlating diameters to characteristic fluorescence lifetimes. (A) Diameter histograms (*n* = 95, 127, 88, and 106) for the four samples (blue, green, yellow, and red) extracted from transmission electron microscopy and the model fits (solid lines). (B) Fluorescence lifetime measurements and the model fits (black solid lines, overlayed on top of the experimental data) for the four samples (blue, green, yellow, and red, vertical rescaling has been performed to avoid occlusion). (C) Estimated joint distributions of diameters and characteristic lifetimes with marginal diameter distributions at the top and marginal characteristic lifetime distributions at the right.

Otherwise, the analysis is identical to the previous one for wavelengths. Fig 2 shows the fluorescence lifetime distributions, the model fits, and the estimated joint distributions of diameters and characteristic lifetimes. The results show that the larger particles have narrower lifetime distributions. This may be due to the presence of fewer defects related to a lower surface-to-volume ratio.

Second, we study the probabilistic relationship between molecular weight *M* and diffusion coefficient *D* for polymers. A well-known empirical scaling law for linear polymer chains states that

$$D\left(M\right)=KM^{-\nu }\Leftrightarrow M\left(D\right)=K^{1/\nu }D^{-1/\nu },$$ (6)

where *ν* ∼ 0.5 − 0.8 for uncharged polymers in the dilute regime, depending on solvent quality and temperature [16]. The parameter *ν*, analogous to the Flory exponent [17], is a measure of how polymers spread in space [18, 19]. Knowledge of both parameters *K* and *ν* allows determination of molecular weight distributions from diffusion coefficient distribution measurements [20]. Polymers comprise a prototypical example of the case where samples are often purified to minimise heterogeneity, typically estimating the scaling law parameters *K* and *ν* by taking logarithms and using linear least-squares on a set of many samples [21]. Considering a polydisperse sample with marginal distributions *p*(*M*) and *p*(*D*), the scaling law relationship can be seen as a degenerate joint distribution with non-zero density only on the line described by Eq 6 because no other parameter influences the relationship, hence it is a special case of the more general example above. We use the fact that if *p*(*M*) is a lognormal distribution with parameter (*μ*_*M*_, *σ*_*M*_), *p*(*D*) is also a lognormal distribution with parameter (*μ*_*D*_, *σ*_*D*_). On a polydisperse polystyrene sample, we measure the molecular weight distribution by gel permeation chromatography (GPC), obtained as a distribution over log_10_ *M*. We measure the diffusion coefficient distribution of the polystyrene, diluted in deuterated chloroform (CDCl_3_), by nuclear magnetic resonance (NMR), obtaining a signal attenuation described by the Stejskal-Tanner equation [22]

$$I\left(b\right)=I_{0}\int_{0}^{\infty }p\left(D\right)\text{exp}\left(-bD\right)dD$$ (7)

for a lognormal distribution *p*(*D*) with parameters (*μ*_*D*_, *σ*_*D*_). From these parameters, the scaling law parameters *K* and *ν* can be obtained by

$$\begin{array}{lll} \nu & = & \frac{\sigma_{D}}{\sigma_{M}}\\ K & = & \text{exp}\left(\mu_{D}+\nu \mu_{M}\right). \end{array}$$ (8)

**Fig 3:**
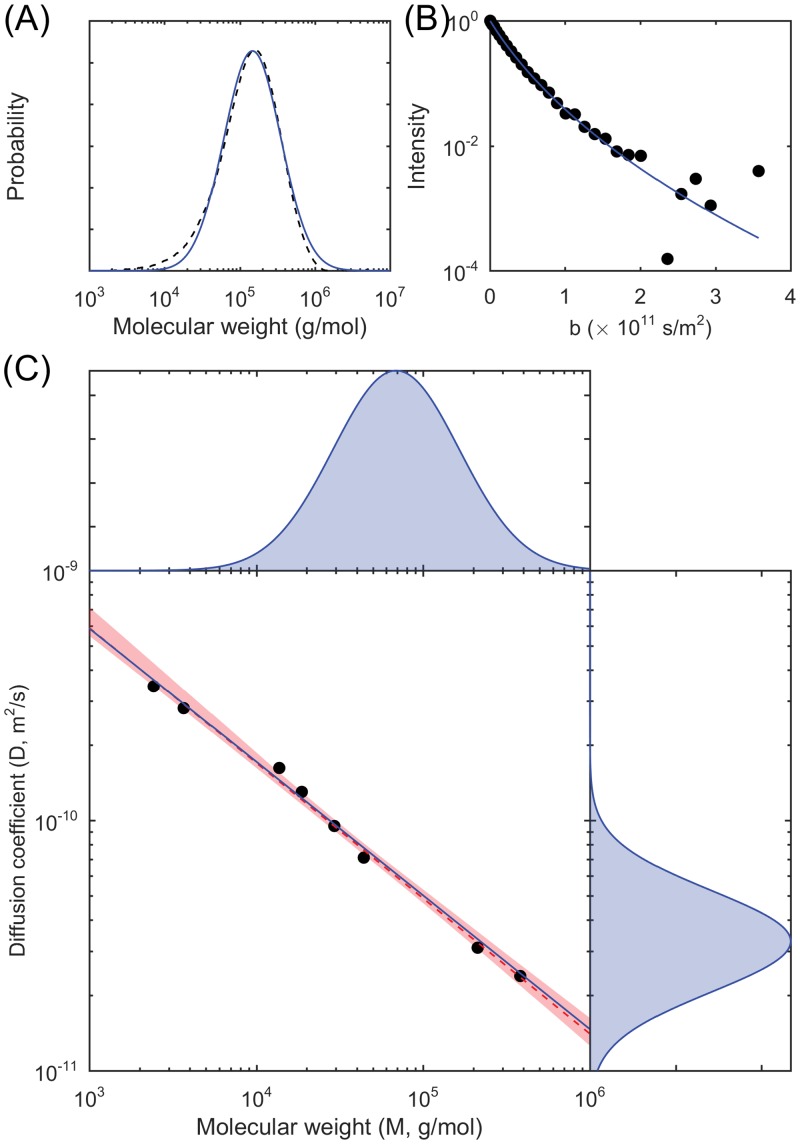
Correlating molecular weights to diffusion coefficients. (A) Molecular weight distribution measured by gel permeation chromatography (dashed black line) and the model fit (solid blue line). (B) Nuclear magnetic resonance attenuation data and the model fit. (C) Estimated joint distribution, degenerating to a distribution on a line, of molecular weight and diffusion coefficient, with the data points for the monodisperse samples (black disks) and the relationship estimated with the conventional method from these samples (dashed red line, with error bounds), relationship estimated with the proposed distribution-based method (solid blue line), and with marginal molecular weight distribution at the top and marginal diffusion coefficient distribution at the right for the polydisperse sample.

As a validation, we use the reported values of M_w_ from the manufacturer for eight well-defined monodisperse polystyrene standards and the corresponding values of 〈*D*〉, measured by NMR for dilute amounts of the samples in CDCl_3_, and perform the conventional linear least-squares fitting to estimate *K* and *ν*. Fig 3 shows the molecular weight distribution data, the diffusion coefficient distribution data, the model fits, and the estimated scaling law relationship. For dilute polystyrene in CDCl_3_ at ambient conditions, we obtain *K* = 2.47 × 10^−8^ and *ν* = 0.541 with the conventional method. Using the proposed distribution-based method, we perform experiments in triplicate and obtain *K* = 2.35 × 10^−8^, 2.67 × 10^−8^, and 2.16 × 10^−8^, and *ν* = 0.534, 0.544, and 0.526. These results are not significantly different from the one obtained with the conventional method based on a 95% nonparametric confidence interval of the latter. That *K* and *ν* can be determined from a single sample has been suggested before [23], but is here performed for the first time.

We emphasise that instead of needing to perform a painstakingly large number of measurements on monodisperse samples (and possibly needing to perform purification steps to obtain those samples), we can obtain a very similar result by different and simpler means, based on performing a single measurement on a polydisperse sample in this case when the functional form of the relationship is known.

## Conclusions

We demonstrate a general-purpose mathematical method for estimating probabilistic relationships from measured distributions of individual parameters. The distributions can be represented directly by statistical samples, or indirectly through an inverse problem by decay curves, spectra, function curves, and histograms. The result is an estimate of a joint probability distribution of parameters that describes relationships and correlations. In the applications to quantum dots, measurements from multiple heterogeneous samples are utilized, allowing for joint probability distributions (Figs 1 and 2) for which the effects of diameter are observed through the dependence of both the mean and the variance of the conditional distribution on diameter. In the application to polymers, using measurements on a single polydisperse polymer sample, the parameters *K* and *ν* of the scaling law relationship in Eq 6 are estimated, and this gives rise to a joint probability distribution (Fig 3) (with conditional distributions being in fact delta functions).

In a world ever more data-rich, with a plethora of measurement techniques and sensors used in the minerals, pharmaceutical, food, and chemical industries, as well as in fundamental science, we urgently need to improve the ways in which we analyse the complex data sets that are becoming the norm. This trend will only continue, in particular because natural raw materials need to be used to their full potential to meet future sustainability and cost demands—materials that are naturally heterogeneous, complex, and challenging to master. We expect our general-purpose mathematical method to be applicable in many fields where heterogeneity and complex distributions of parameters are obstacles to scientific insight. Single values of parameters can be replaced by distributions, taking advantage of the heterogeneity and letting it work for us, not against us. Naturally, multi-dimensional experiments deliver a wealth of information which is literally impossible to access with lower-dimensional experiments. Though more complicated parameter relationships, involving e.g. non-monotonicity, discontinuity, or multi-modal conditional distributions, cannot be resolved with our method, we expect the method to be useful for the understanding of many heterogeneous systems.

## Materials and Methods

### Preparation of quantum dots

Four samples of ZnS-coated CdSe core-shell quantum dots (QDs) with different nominal diameters in the range *d* ≈ 2 − 5 nm are synthesized using a previously published method [24] and dissolved in toluene.

### Transmission electron microscopy measurements

Diameters of QDs are estimated using transmission electron microscopy (TEM). The analysis is performed on a JEOL 2100F microscope operated at 200 kV. All experiments are carried out at room temperature. The spatial resolutions (physical pixel sizes) are Δ*x* = 0.05 − 0.17 nm for the image data. Particle contours (*n* = 95, 127, 88, and 106) for the four samples are identified manually from raw TEM images.

### Luminescence measurements

Steady-state emission spectra of the QDs are acquired in the range 450–650 nm with resolution Δ*λ* = 1 nm using an Edinburgh Photonics FLS 980 time-resolved photoluminescence spectrometer. At each respective emission intensity maxima, fluorescence lifetime measurements by time-correlated single photon counting (TCSPC) are performed using the same instrument. Excitation lifetimes are recorded in a 500 ns range using 8192 channels.

### Nuclear magnetic resonance measurements

Polydisperse polystyrene (PS) (190,000 M_w_, Sigma-Aldrich) is mixed to 0.02% w/w in CDCl_3_ (99.8 atom% deuterium, Sigma-Aldrich). Monodisperse PS standards (2,430 M_w_, 3,680 M_w_, 13,700 M_w_, 18,700 M_w_, 29,300 M_w_, 44,000 M_w_, 212,400 M_w_, and 382,100 M_w_, Sigma-Aldrich) are each mixed to 0.1% w/w, also in CDCl_3_. These concentrations are chosen in order to be within the dilute polymer regime (18) and to avoid microscopic averaging effects [25]. NMR tubes are filled with the PS solutions and flame-sealed to avoid convection due to solvent evaporation. Pulsed Gradient Stimulated Echo (PGSTE) measurements of 1^H^ self-diffusion [26] are performed at ambient conditions using a Bruker Avance III HD NMR spectrometer with an Ascend 600 MHz superconducting magnet, a micro5 probe, and a diff30 gradient coil. The PGSTE parameters for all measurements are repetition time TR = 10 s, gradient pulse duration *δ* = 1.576 ms, observation time Δ = 50 ms, and *τ* = 2.6 ms. The PGSTE measurements on the 0.02% w/w 190,000 M_w_ polydisperse PS sample, performed in triplicate, use 192 averages and 32 linearly spaced gradient steps with maximum gradient value 10 T/m. The PGSTE measurements on the PS standard samples use 16 averages and 16 linearly spaced gradient steps with maximum gradient values chosen to attenuate 30% of the PS signals. The spectrally resolved PS signal attenuations are analysed using the Stejskal-Tanner equation, Eq 7, with attenuation factor

$$b={\left(\gamma g\delta \right)}^{2}\frac{4}{\pi^{2}}\left(\Delta -\frac{\delta }{4}\right),$$ (9)

for a sinusoidal gradient pulse shape.

### Gel permeation chromatography measurements

Gel permeation chromatography (GPC) molecular weight distribution measurements of the 190,000 M_w_ polydisperse PS are performed on an Agilent PL-GPC 220 system flowing trichlorobenzene at 1 ml/min through three PLgel 10 *μ*m mixed-B columns at 150°C. Measurements are performed in triplicate on 1.5 mg/ml samples using a 200 *μ*l injection volume and conventional calibration with Agilent EasiVial PS-H standards. The data is obtained as 400–500 points equidistant in log_10_ *M*.

### Data analysis for quantum dots

All particle contours identified from the TEM images are near-circular, and an equivalent diameter is computed by

$$d={\left(\frac{4A}{\pi }\right)}^{1/2}=2{\left(\frac{N}{\pi }\right)}^{1/2}\Delta x,$$ (10)

where *A* is area and *N* is number of pixels within the contour. We make parametric model assumptions and fit lognormal distributions,

$$p_{d;k}\left(d\right)=\frac{1}{d\sigma_{d,k}\sqrt{2\pi }}\text{exp}\left(-\frac{{\left(\text{log}\left(d\right)-\mu_{d,k}\right)}^{2}}{2\sigma_{d,k}^{2}}\right),$$ (11)

to the diameters using maximum likelihood [27], resulting in parameter estimates

$$\begin{array}{lll} \mu_{d,k} & = & \frac{1}{n}\underset{i}{\sum }\text{log}\left(d_{i}\right)\\ \sigma_{d,k} & = & \frac{1}{n}\underset{i}{\sum }{\left(\text{log}\left(d_{i}\right)-\mu_{d,k}\right)}^{2}. \end{array}$$ (12)

Steady-state emission spectra are interpreted as probability distribution of wavelengths convolved with a Lorentzian curve (Cauchy distribution with parameter *γ*, corresponding to a FWHM of 2*γ*.), accounting for line broadening. All spectra are fit simultaneously using nonlinear least-squares, thus simultaneously estimating *p*_λ|*d*_(*d*, λ) and *p*_λ;*k*_(λ) for fixed *p*_*d*;*k*_(*d*) by minimizing the global sum of squares

$$\text{SS}=\sum_{k}\sum_{l}{\left(I_{k}\left(\lambda_{l}\right)-I_{k}^{\text{exp}}\left(\lambda_{l}\right)\right)}^{2},$$ (13)

summed over all spectra and all wavelengths. For fluorescence lifetime measurements by TCSPC, time zero is determined by finding the channel with the highest photon count. The lifetime distributions are interpreted as superpositions of exponential distributions,

$$p_{t,k}\left(t\right)=\int_{0}^{\infty }p_{\tau ;k}\left(\tau \right)\frac{1}{\tau }\text{exp}\left(-\frac{t}{\tau }\right)\;\text{d}\tau .$$ (14)

governed by ‘characteristic’ (average) lifetime distributions *p*_*τ*;*k*_(*τ*). Data is acquired in discrete time, as a histogram, and fitting of the discrete-time counterpart of Eq 14 with an additional baseline (dark photon count) probability is performed using maximum likelihood and ‘discretized’ channel probabilities, corresponding to the histogram binning, *q*_*k*, *l*_ for bin *l*. For further details on fitting refer to Röding (2014) [28]. Because the photon counts differ between lifetime measurements (between *N* = 2.6 × 10^5^ − 2.1 × 10^6^), weighted maximum likelihood is employed. All lifetime distributions are fit simultaneously using weighted maximum likelihood, thus simultaneously estimating *p*_*τ*|*d*_(*d*, *τ*) and *p*_*τ*;*k*_(*τ*) for fixed *p*_*d*;*k*_(*d*) by maximizing the weighted global loglikelihood function

$$\text{log}L=\sum_{k}\sum_{l}\frac{n_{k,l}}{N_{k}}\text{log}q_{k,l},$$ (15)

summed over all lifetime distributions and all bins, and where *n*_*k*, *l*_ is the number of photons in bin *l* of distribution *k*, and *N*_*k*_ is the total number of photons in distribution *k*. Integrals in (*d*, λ) and (*d*, *τ*) space are computed using monte carlo integration with *n*_mc_ = 10^4^ quasi-random samples [29]. As a model for *p*_λ|*d*_(*d*, λ) we choose a lognormal distribution with diameter-dependent mean *m*_λ_(*d*) and standard deviation *s*_λ_(*d*). We let both *m*_λ_(*d*) and *s*_λ_(*d*) be power-law type functions,

$$m_{\lambda }\left(d\right)=m_{1}+m_{2}d^{m_{3}}$$ (16)

and

$$s_{\lambda }\left(d\right)=s_{1}+s_{2}d^{s_{3}}.$$ (17)

Data analysis is performed using Matlab R2015a (Mathworks, Natick, MA, US).

### Data analysis for polymers

Molecular weight distributions measured by GPC are fit using a normal distribution in log_10_ *M* space with parameters (*μ*_*M*_/log10, *σ*_*M*_/log10), using nonlinear least-squares and minimizing

$$\text{SS}=\sum_{i}{\left(p_{\text{GPC}}\left({\text{log}}_{10}M_{i}\right)-p_{\text{Normal}}\left({\text{log}}_{10}M_{i};\frac{\mu_{M}}{\text{log}10},\frac{\sigma_{M}}{\text{log}10}\right)\right)}^{2}.$$ (18)

By a well-known scaling property of the lognormal distribution family, the scaling law in Eq (6) implies that *D* is also lognormal distributed with parameters

$$\begin{array}{lll} \mu_{D} & = & \text{log}K-\nu \mu_{M}\\ \sigma_{D} & = & \nu \sigma_{M}. \end{array}$$ (19)

This derivation is fairly straightforward by use of the standard change-of-variables technique for probability distributions. Hence, a lognormal distribution model for *p*(*D*) is used in the Stejskal-Tanner equation in Eq (7), using numerical integration with *n*_*grid*_ = 10^3^ grid points to compute the values of the signal attenuation. The model is fit to the experimental NMR signal attenuation using nonlinear least-squares [20].

Bootstrapping [30] is used for obtaining confidence bounds for the conventionally estimated scaling law relationship. The eight measurement points are resampled with replacement *n*_boot_ = 10^5^ times, and 2.5% and 97.5% percentiles are computed pointwise along the *M* axis to yield a 95% nonparametric confidence interval.

Data analysis is performed using Matlab R2015a (Mathworks, Natick, MA, US).

## Supporting Information

S1 Code and Data Sets Matlab programs and data sets used in this study. (ZIP)
